# Astragalin Promotes Osteoblastic Differentiation in MC3T3-E1 Cells and Bone Formation *in vivo*

**DOI:** 10.3389/fendo.2019.00228

**Published:** 2019-04-16

**Authors:** Li Liu, Dan Wang, Yao Qin, Maolei Xu, Ling Zhou, Wenjuan Xu, Xiaona Liu, Lei Ye, Shijun Yue, Qiusheng Zheng, Defang Li

**Affiliations:** ^1^School of Integrated Traditional Chinese and Western Medicine, Binzhou Medical University, Yantai, China; ^2^School of Pharmacy, Guangdong Medical University, Dongguan, China; ^3^School of Pharmacy, Binzhou Medical University, Yantai, China; ^4^Key Laboratory of Xinjiang Endemic Phytomedicine Resources, Ministry of Education, School of Pharmacy, Shihezi University, Shihezi, China

**Keywords:** MC3T3-E1 cells, osteoblastic differentiation, Astragalin, BMP-2, MAPK, bone formation

## Abstract

Astragalin (AG) is a biologically active flavonoid compound that can be extracted from a number of medicinal plants. However, the effects of AG on osteoblastic differentiation in mouse MC3T3-E1 cells and on bone formation *in vivo* have not been studied fully. In this study, we found that the activities of alkaline phosphatase (ALP) and mineralized nodules in MC3T3-E1 cells were both significantly increased after treatment with AG (5, 10, and 20 μM). Meanwhile, the mRNA and protein levels of osteoblastic marker genes in MC3T3-E1 cells after AG treatment were markedly increased compared with a control group. In addition, the levels of BMP-2, p-Smad1/5/9, and Runx2 were significantly elevated in AG-treated MC3T3-E1 cells. Moreover, we found that the protein levels of Erk1/2, p-Erk1/2, p38, p-p38, and p-JNK were also significantly increased in AG-treated MC3T3-E1 cells compared to those in the control group. Finally, *in vivo* experiments demonstrated that AG significantly promoted bone formation in an ovariectomized (OVX)-induced osteoporotic mouse model. This was evidenced by significant increases in the values of osteoblast-related parameters (BFR/BS, MAR, Ob.S/BS, and Ob.N/B.Pm) and bone histomorphometric parameters (BMD, BV/TV, Tb.Th, and Tb.N.) in OVX mice after AG treatment (5, 10, and 20 mg/kg). Collectively, these results demonstrated that AG may promote osteoblastic differentiation in MC3T3-E1 cells via the activation of the BMP and MAPK pathways and promote bone formation *in vivo*. These novel findings indicated that AG may be a useful bone anabolic agent for the prevention and treatment of osteoporosis.

## Introduction

Osteoporosis is a systemic skeletal disease characterized by global damage to bone mass and an enhanced risk of bone fractures (1). Clinical data have shown that the vast majority of osteoporosis patients not only endure significant pain for long periods of time, but also are subject to a great financial burden as treatment options are costly (2). Indeed, it has been reported that one of the leading causes of increased health care costs in the United States is osteoporosis (3). For the aging population, the medical and socioeconomic impacts of osteoporosis, particularly for postmenopausal osteoporosis, are expected to continue to increase (4). Therefore, the prevention and treatment of osteoporosis are urgent medical questions that needs to be answered. Typically, the maintenance of bone homeostasis requires coordinated work by both bone-forming osteoblasts and bone-resorbing osteoclasts (5). However, osteoporosis occurs as a result of decreased bone formation induced by osteoblasts and increased bone resorption induced by osteoclasts. Recent studies have suggested that stimulation of osteoblast differentiation may prove to be an efficacious treatment strategy for the prevention and treatment of osteoporosis (6).

Runt-related transcription factor 2 (Runx2), a major transcription factor, is required for the activation of osteoblast differentiation and the expression of the bone formation-related genes ALP, OCN, and OPN. Bone morphogenetic protein (BMP), an important member of the transforming growth factor-β (TGF-β) superfamily, has been shown to target Runx2 to induce bone formation by stimulating osteoblastic differentiation in mesenchymal stem cells (7). In addition, BMP-2 can bind BMP receptors to induce heteromeric complexes and the phosphorylation of Smad1/5/9, subsequently inducing osteoblast differentiation (8). Moreover, Runx2 is also a downstream molecule of the mitogen-activated protein kinase (MAPK) signaling pathway during osteoblast differentiation (9). MAPKs are a family of serine/threonine kinases that play significant roles in different cellular processes, such as proliferation, differentiation, and inflammation. It has been reported that the activation of the p38 MAPK and Erk1/2 signaling pathway can up-regulate bone formation-related gene expression and promote osteoblastic differentiation and mineralization (10).

Flavonoids are polyphenolic compounds widely distributed in fruits, vegetables, and botanical drugs. They possess a number of potent biological effects and are anti-inflammatory, antioxidant, and anti-carcinogenic (11). Astragalin (AG), also called kaempferol-3-O-glucoside, is a flavonoid compound extracted from various traditional herbs and medicinal plants (11). Studies have demonstrated that AG has anti-atopic dermatitis and anti-mastitis effects and acts as an antioxidant, as well as an anti-inflammatory agent (12, 13). A recent study indicated that out of five known flavonoids, AG may regulate the activity of ALP in osteoblast-like UMR-106 cells, implying AG may have osteogenic effects (14). Therefore, in this study, we evaluated whether AG induces osteoblastic differentiation in MC3T3-E1 cells and whether it promotes bone formation in a mouse model of osteoporosis.

## Materials and Methods

### Reagents and Chemicals

AG (chemical formula: C_21_H_20_O_11_, molecular weight: 448.38, purity ≥ 98%) was obtained from Beijing Bailingwei Technology Co. Ltd. (Beijing, China). AG was first dissolved in dimethyl sulphoxide (DMSO), followed by dilution with α-Minimum Essential Medium (α-MEM) to achieve the appropriate concentrations. The final concentration of DMSO in the complete culture medium was no >0.1%. Fetal bovine serum (FBS) and α-MEM were purchased from Thermo Fisher Scientific (Waltham, MA, USA). Alizarin red S staining solution, 4% paraformaldehyde, penicillin and streptomycin, and Trizol reagent were purchased from Solarbio Science & Technology Co. Ltd. (Beijing, China). The alkaline phosphatase assay kit used in this study was obtained from Beyotime Biotechnology Co. Ltd. (Guangzhou, China). Unless otherwise indicated, all other chemical reagents were obtained from Sigma (St. Louis, MO, USA).

### Cell Culture

MC3T3-E1 cells were obtained from the Cell Bank of the Committee on Type Culture Collection of the Chinese Academy of Sciences (Shanghai, China). MC3T3-E1 cells were cultured in a cell incubator with 5% CO_2_ at 37°C, and were maintained in α-MEM complete culture medium containing 10% FBS, 100 μg/mL streptomycin, and 100 U/mL penicillin.

### Cell Viability Assay

The effects of AG on MC3T3-E1 cell viability were measured by the MTT assay. Briefly, MC3T3-E1 cells were digested with 0.05% Trypsin-EDTA and subsequently collected after centrifugation. Then the cells were cultured in 96-well plates at ~7 × 10^3^ cells per well and incubated in the cell incubator. After 24 h of incubation, six different concentrations of AG (5, 10, 20, 30, 40, and 50 μM) were added every 24 h. After treatment for 48 h, 10 μL of MTT solution (5 mg/mL in PBS, pH 7.4) was added to each well, and plates were placed in the cell incubator for 4 h at 37°C. Subsequently, the culture supernatant was carefully discarded and DMSO (150 μL/well) was added to the plates. Next, the plates were vibrated on a shaker for 10 min to dissolve the formazan crystal violet completely. Then a microplate reader (Bio-Rad Laboratories Inc., Hercules, CA, USA) was used to determine the absorbance of each well at 490 nm. Cellular proliferation was assessed by calculating and comparing the relative survival rate of the cells between the control and experimental groups (15).

### Alkaline Phosphatase Activity Assay

The cells were incubated in 6-well plates at a density of 2 × 10^5^ cells per well. To induce osteoblastic differentiation, all the groups (except the blank group) were cultured with osteogenic supplement (OS: 50 μg/mL ascorbic acid, 10 μM dexamethasone, and 5 mM β-glycerophosphate in the complete α-MEM) with slight changes as previously described (16). The cells in the blank group were treated with complete α-MEM containing 0.1% DMSO. The cells in the control group were cultured only with α-MEM containing 0.1% DMSO and osteogenic supplement (OS induction). The experimental groups were also exposed to three different concentrations of AG (5, 10, and 20 μM) for 7 or 14 days; the concentrations of AG were added every 24 h, and the culture medium was exchanged with fresh medium every 3 days. Next, the cells were lysed with a cell lysis solution, and the activity of alkaline phosphatase was determined with the Alkaline Phosphatase Assay Kit according to the manufacturer's instructions. Then, the microplate reader (Bio-Rad Laboratories Inc.) was used to detect the absorbance of each group at 405 nm.

### Alizarin Red Staining

The cells were incubated in 6-well plates at a density of 2 × 10^5^ cells per well. All the groups (except the control group) were cultured with OS. The experimental groups were also exposed to three different concentrations of AG (5, 10, and 20 μM) for 14 or 21 days. The three different concentrations of AG were added every day. Then, the original culture medium was discarded and the AG-treated cells were gently washed with PBS twice, followed by fixation in 4% paraformaldehyde solution. After 20 min, the 4% paraformaldehyde solution was discarded and the cells were washed with PBS twice. Subsequently, the Alizarin Red S staining solution was used to stain the cells for 30 min. Then the cells were washed with PBS and allowed to dry naturally. The cells were then observed and photographed using a microscope. Five images were captured per well at 40-fold magnification (triplicate wells per group). Professional image analysis software (Image J, NIH, Bethesda, MA, USA) was used to quantify the mineralized nodules, and the mineralized modules whose areas exceeded 0.04 mm^2^ were counted (17).

### RT-qPCR Assay

The cells were incubated in 6-well plates at a density of 2 × 10^5^ cells per slide; the cell culture methods were the same as above. Total RNA was isolated and purified using Trizol (Solarbio Science & Technology Co. Ltd., Beijing, China) according to the manufacturer's instructions. Then a RevertAid First Strand cDNA Synthesis Kit (Cat no. K1622, Thermo Scientific) was used to reverse-transcribe 1 μg of purified RNA in a 20 μL volume into cDNA. The thermocycler parameters were as follows: for 10 min; 40 cycles of for 10 s, 56°C for 30 s, and for 20 s. The primer sequences used for PCR were as follows: Mouse β-actin primers: forward primer 5′-AGGTCGGTGTGAACGGATTTG-3′ and reverse primer 5′-TGTAGACCATGTAGTTGAGGTCA-3′; Mouse ALP primers: forward primer 5′-AACCCAGACACAAGCATTCC-3′ and reverse primer 5′-GAGAGCGAAGGGTCAGTCAG-3′; Mouse OCN primers: forward primer 5′-AAGCAGGAGGGCAATAAGGT-3′ and reverse primer 5′-TTTGTAGGCGGTCTTCAAGC-3′; Mouse OPN primers: forward primer 5′-AGCAAGAAACTCTTCCAAGCAA-3′ and reverse primer 5′-GTGAGATTCGTCAGATTCATCCG-3′; Mouse Runx2 primers: forward primer 5′-ACTCTTCTGGAGCCGTTTATG-3′ and reverse primer 5′-GTGAATCTGGCCATGTTTGTG-3′; Mouse BMP-2 primers: forward primer 5′-ACACAGCTGGTCACAGATAAG-3′ and reverse primer 5′-CTTCCGCTGTTTGTGTTTGG-3′. The relative expression levels of the above genes were calculated according to the following formula: 2^−ΔΔCT^ and ΔΔC_T_ = ΔC_T_(X) – ΔC_T_(Y), where X represents the AG-treated groups and Y represents the control group. Mouse β-actin was used as internal control (18). Data Sheet 1 for the raw data for all the real-time PCR analysis.

### Western Blot Analysis

AG-treated MC3T3-E1 cells were washed with PBS and lysed with RIPA lysis buffer. After centrifugation (12,000 × g at 4°C) for 15 min, the supernatants containing protein (the protein samples) were collected. The protein concentrations of the samples were measured by an enhanced BCA protein assay kit (Solarbio Science & Technology Co. Ltd., Beijing, China) according to the manufacturer's instructions. Then, the protein samples were added to 5X sample buffer and boiled to denature the proteins. The proteins were, then, separated by SDS-polyacrylamide gel electrophoresis at constant 100 V-pressure and, subsequently, blotted onto nitrocellulose membranes (Amersham Biosciences, Piscataway, NJ, USA) at 250 mA for 1.5 h. The membranes were blocked with 5% BSA in TBST buffer for 2 h at room temperature, followed by incubation at 4°C with the primary antibodies as follows: anti-β-actin (Abcam, ab179467, 0.14 μg/mL), anti-ALP (Abcam, ab229126, 1 μg/mL), anti-OPN (Abcam, ab33046, 1.25 μg/mL), anti-OCN (Abcam, ab93876, 2 μg/mL), anti-BMP-2 (Abcam, ab14933, 1 μg/mL), anti-p-Smad1/5/9 (Cell Signaling Technology, #13820, 1 μg/mL), anti-Runx2 (Abcam, ab23981, 1 μg/mL), anti-Erk1/2 (Abcam, ab17942, 0.5 μg/mL), anti-p-Erk1/2 (Cell Signaling Technology, #4370, 0.5 μg/mL), anti-JNK (Abcam, ab179461, 1.75 μg/mL), anti-p-JNK (Abcam, ab124956, 0.75 μg/mL), anti-p38 (Abcam, ab170099, 0.25 μg/mL), and anti-p-p38 (Abcam, ab47363, 1 μg/mL). Subsequently, the membranes were washed with TBST buffer and were incubated with TBS buffer containing secondary antibodies for 1 h at room temperature. Finally, the membranes were incubated with enhanced ECL chemiluminescence detection solution (Thermo Fisher, NY, USA), and image information was obtained using a UVP chemiluminescence imaging system (18, 19). Data Sheet 2 depicts uncropped western blots.

### OVX-Induced Osteoporotic Mouse Model

Thirty-two female C57BL/6 mice (12 weeks old) were used in this study and were maintained in the animal room at Binzhou Medical University. The mice were provided a commercial standard mouse diet and water *ad libitum*. The room was kept at a temperature of 25°C with a 12 h light/12 h dark cycle. Female mice were ovariectomized (OVX) according to our previous study (20). Then OVX mice were randomly grouped into four sub-groups to receive the following treatments: (1) OVX group (model group, *n* = 8), (2) OVX mice administrated AG (5 mg/kg per day) via intraperitoneal injection (i.p.) [OVX + AG(5), *n* = 8], (3) OVX mice administered AG, 10 mg/kg per day, i.p. [OVX + AG(10), *n* = 8], (4) OVX mice administered AG, 20 mg/kg per day, i.p. [OVX + AG(20), *n* = 8]. The mice were administered the different concentrations of AG for 4 weeks. Meanwhile, all the mice were administered xylenol orange (90 mg/kg) and calcein green (10 mg/kg) at 10 and 2 days through intraperitoneal injection, respectively, before euthanasia. After sacrifice, the bilateral femora were isolated and collected. The Committees of Animal Ethics and Experimental Safety of Binzhou Medical University approved all the experimental procedures used in this study, which conforms with NIH guidelines for the care and use of laboratory animals. In this *in vivo* study, selected doses (5, 10, and 20 mg/kg) of AG were based on the concentrations used in *in vitro* experiments and the doses used in mice in a previous study (21).

### Micro-CT

The bilateral femora isolated from the AG-treated mice were scanned with a micro-CT system (Viva CT40, SCANCO Medical, Switzerland). Briefly, a total of 200 slices with a voxel size of 15 μm were detected above the growth plate of the distal femur. The trabecular bone was selected for three-dimension reconstruction (Sigma = 1.2, Threshold = 180, and Supports = 2) to calculate the following parameters: trabecular thickness (Tb.Th.), bone mineral density (BMD), relative bone volume (BV/TV), and trabecular number (Tb.N.).

### Bone Histomorphometry

The distal femurs isolated from the AG-treated mice were fixed in a 4% paraformaldehyde solution and subsequently dehydrated with ethanol. After dehydration, femurs were embedded without decalcification in modified methyl methacrylate (MMA) using our previously established procedures (20). Then, the dehydrated femurs were cut into a thickness of 15 mm using a Leica SP1600 microtome (Leica Microsystems, Germany). The fluorescence intensities of the xylenol orange and calcein green in the anterior part of trabecular bone were, then, detected by fluorescence microscopy (Q500MC, Leica Microsystems, Germany). Meanwhile, modified Masson's trichrome staining and tartrate-resistant acid phosphatase (TRAP) staining were employed to stain the bone sections. Bone dynamic histomorphometric parameters [mineral apposition rate (MAR) and bone formation rate (BFR/BS)] and bone static histomorphometric parameters [osteoblast surface (the percent of trabecular bone surface covered by osteoblasts, Ob.S/BS), osteoblast number per bone perimeter (Ob.N/B.Pm), osteoclast surface (the percent of trabecular bone surface covered by osteoclasts, Oc.S/BS), and osteoclast number per bone perimeter (Oc.N/B.Pm)] were calculated using professional image analysis software (Image J) according to the standardized nomenclature and units for bone histomorphometry.

### Modified Masson's Trichrome Staining

The isolated femurs were fixed in 4% formaldehyde buffer for 24 h and decalcified with 10% EDTA for 4 weeks. Then, the femurs were dehydrated and embedded in paraffin. Serial 5-μm-thick sections were then cut and coated on slides. The slides were rehydrated with 100, 95, 70, and 50% alcohol solutions. After rehydration, the slides were washed in distilled water and placed in Bouin's solution for 15 min at 56°C. Then, the slides were rinsed in running tap water for 5 min to discard the picric acid (yellow color). After rinsing, the slides were counterstained with Weigert's working hematoxylin for 10 min and washed in running tap water for 5 min, followed by rinsing three times with distilled water. Then the slides were stained with Biebrich scarlet acid fuchsin for 5 min and rinsed three times with distilled water. In the next step, the slides were immersed in phosphotungstic/phosphomolybdic acid for 10 min and transferred to the aniline blue solution for 5 min. Finally, the slides were washed with distilled water and differentiated in 1% acetic acid for 1 min. After dehydration and mounting, the stained bone sections were observed using a microscope (Q500MC, Leica Microsystems, Germany) and were analyzed by professional image analysis software (Image J).

### TRAP Staining

After fixation and decalcification, the femurs were dehydrated and embedded in paraffin. Then, the femurs were cut into 5-μm-thick sections, which were coated on slides and rehydrated with 100, 95, 70, and 50% alcohol solutions. The slides were, then, stained using a TRAP staining kit (Sigma-Aldrich, Cat no. 387A-1KT) according to the manufacturer's protocol. After dehydration and mounting, the stained slides were observed under a microscope (Q500MC, Leica Microsystems, Germany) and were analyzed by professional image analysis software (Image J).

### Statistical Analysis

Each cell experiment in this study was repeated at least three times; all the experimental data are represented as the mean ± standard deviation (SD) and were analyzed using SPSS 21.0 statistical software. Statistical analyses were performed using the two-tailed Student's *t*-test or one-way analysis of variance (ANOVA), followed by the least significant difference (LSD) test for data with a normal distribution or the Kruskal–Wallis test for data not normally distributed. *P* < 0.05 was considered statistically significant.

## Results

### AG Induces Osteoblastic Differentiation in Mouse MC3T3-E1 Cells

**Figure 1:**
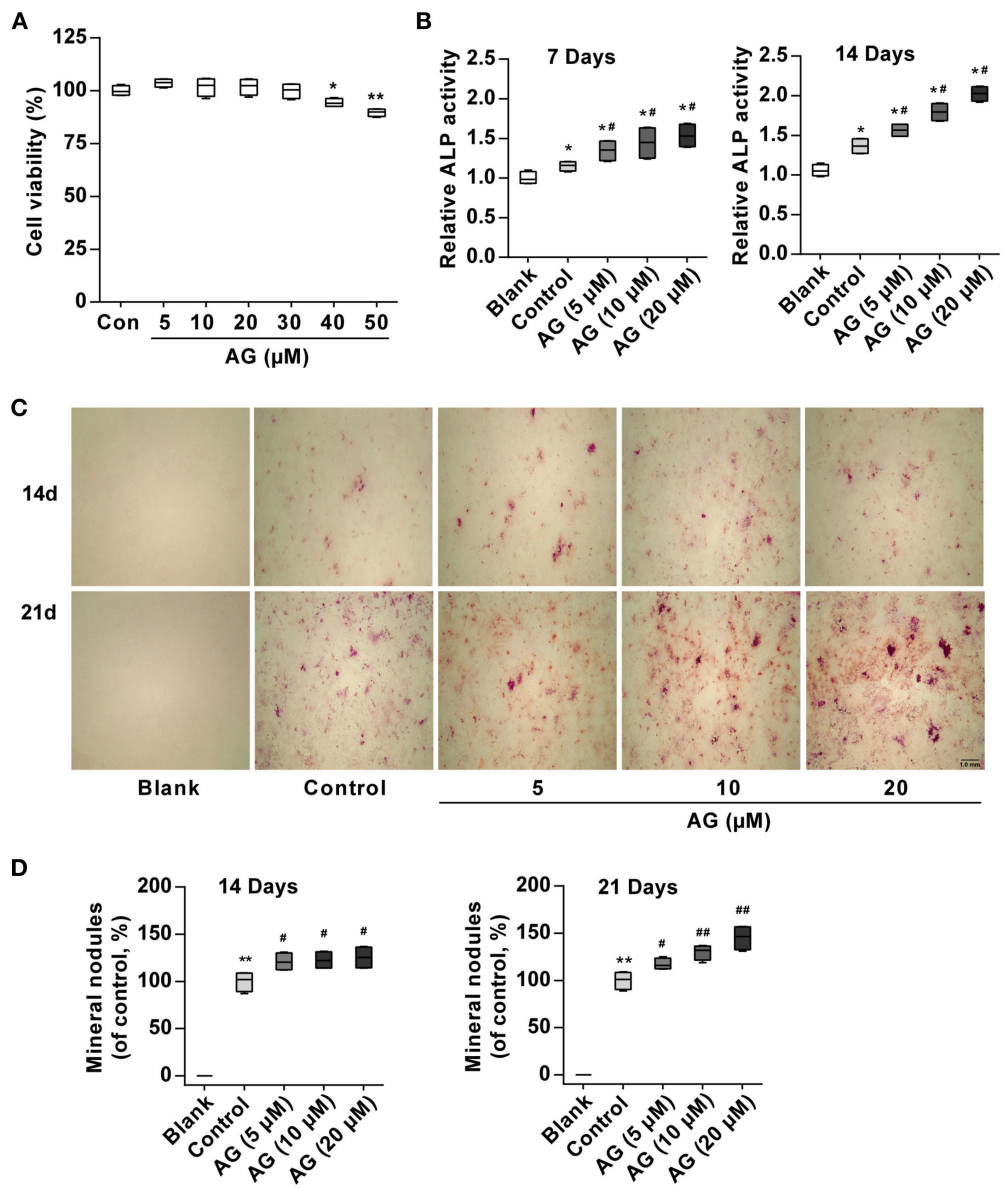
The effects of AG on osteoblastic differentiation and matrix mineralization in mouse MC3T3-E1 cells. **(A)** Viability of MC3T3-E1 cells was measured by MTT assay after 48 h of AG treatment. ^*^P < 0.05, ^**^P < 0.01 compared with the control group. *N* = 4. **(B)** ALP activity was determined by using the Alkaline Phosphatase Assay Kit after 7 and 14 days of AG treatment. ^*^P < 0.05 compared with the corresponding blank group. ^#^*P* < 0.05 compared with the corresponding control group. *N* = 4. **(C)** Representative images of mineralized nodules after Alizarin Red S staining. Scale bar, 1.0 mm. **(D)** Quantitative analysis of mineralized nodules after Alizarin Red S staining. ^*^P < 0.05 compared with the corresponding blank group. ^#^*P* < 0.05, ^##^*P* < 0.01 compared with the corresponding control group. *N* = 4. All of the data are shown as the mean ± S.D. of the independent experiments.

To observe whether AG induces osteoblastic differentiation in mouse MC3T3-E1 cells, its effect on cell growth was first observed. MTT assays showed that AG was not cytotoxic to cells up to 30 μM (Figure 1A). Subsequently, to avoid the cytotoxic effect of AG on MC3T3-E1 cells, we selected 5, 10, and 20 μM concentrations of AG for the following experiments. ALP is a major marker and an essential enzyme in the early stage of osteoblastic differentiation. In addition, the extracellular matrix gradually is mineralized because of calcium deposition and eventually forms bone nodules in the late stage (22, 23). Thus, we examined the activity of ALP and mineralized nodules and mRNA/protein levels of bone formation-related genes. Our results demonstrated that AG significantly elevated ALP activity in a concentration-dependent manner (Figure 1B). Meanwhile, Alizarin Red S staining demonstrated that AG increased the matrix mineralization of mouse MC3T3-E1 cells in a concentration- and time-dependent manner (Figures 1C,D).

### AG Stimulates Higher Expression of Osteoblastic Marker Genes in MC3T3-E1 Cells

**Figure 2:**
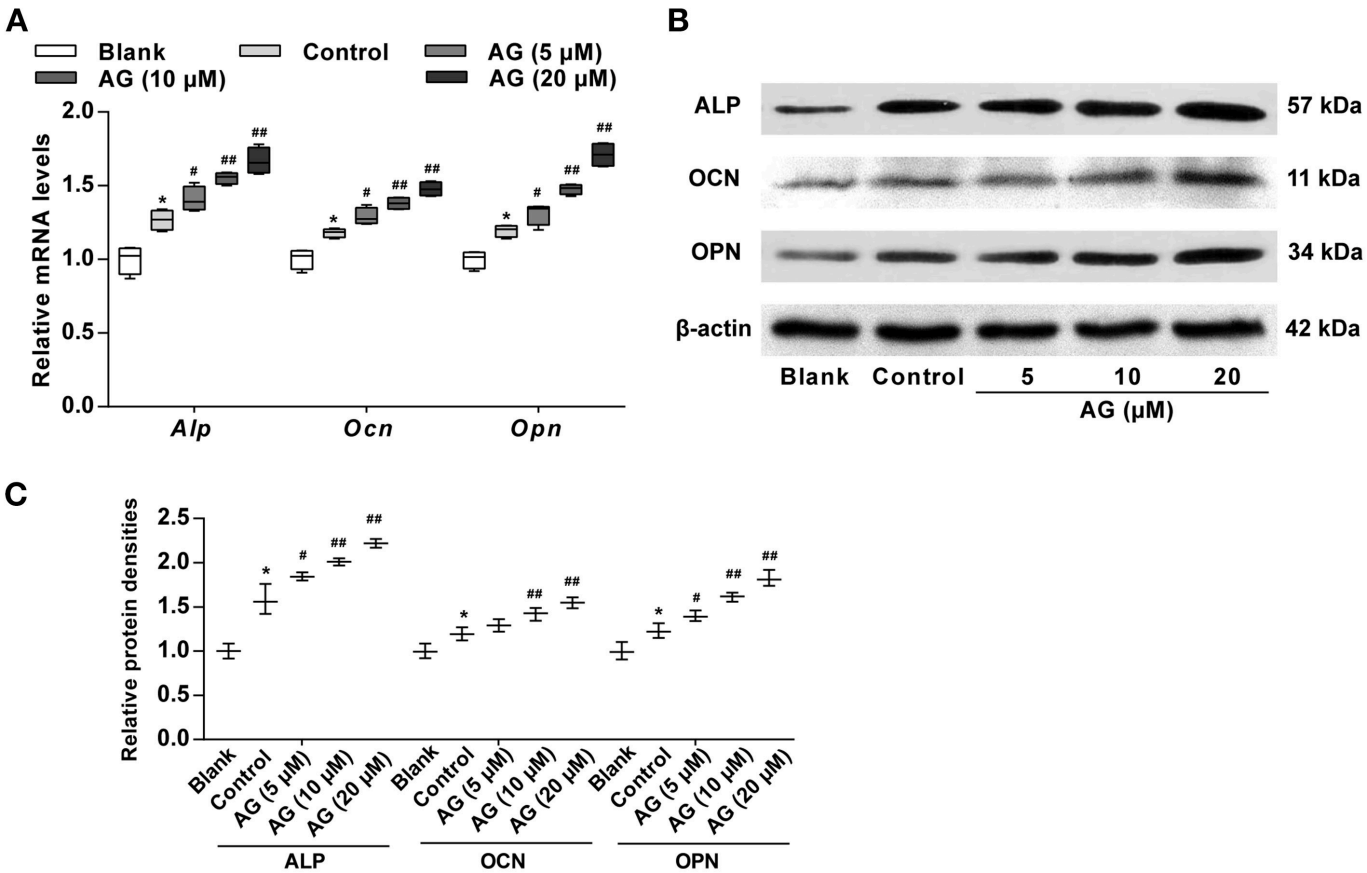
The effects of AG on the expression of osteoblastic marker genes in mouse MC3T3-E1 cells. **(A)** Quantitative analysis of the mRNA levels of *Alp, Ocn*, and *Opn* in MC3T3-E1 cells after treatment with AG for 7 days was determined by RT-qPCR. *N* = 4. **(B)** The protein levels of ALP, OCN, and OPN in MC3T3-E1 cells after treatment with AG for 7 days were measured by western blot. **(C)** Quantitative analysis of the protein level of ALP, OCN, and OPN in MC3T3-E1 cells after treatment with AG for 7 days. *N* = 3. All of the data are shown as the mean ± S.D. of the independent experiments. ^*^P < 0.05 compared with the corresponding blank group. ^#^*P* < 0.05, ^##^*P* < 0.01 compared with the corresponding control group.

Next, RT-qPCR and western blot analysis were employed to measure mRNA and protein levels of osteoblastic marker genes, including *Alp, Ocn*, and *Opn* in AG-treated MC3T3-E1 cells. When compared with the control group (OS induction), the mRNA levels of these genes in MC3T3-E1 cells were significantly increased after AG (10 and 20 μM) treatment (Figure 2A). The protein levels of the above maker genes were also significantly up-regulated in AG-treated groups (10 and 20 μM) when compared with the corresponding control group (Figures 2B,C).

### AG Up-Regulates the Expression of BMP Signaling Molecules in MC3T3-E1 Cells

**Figure 3:**
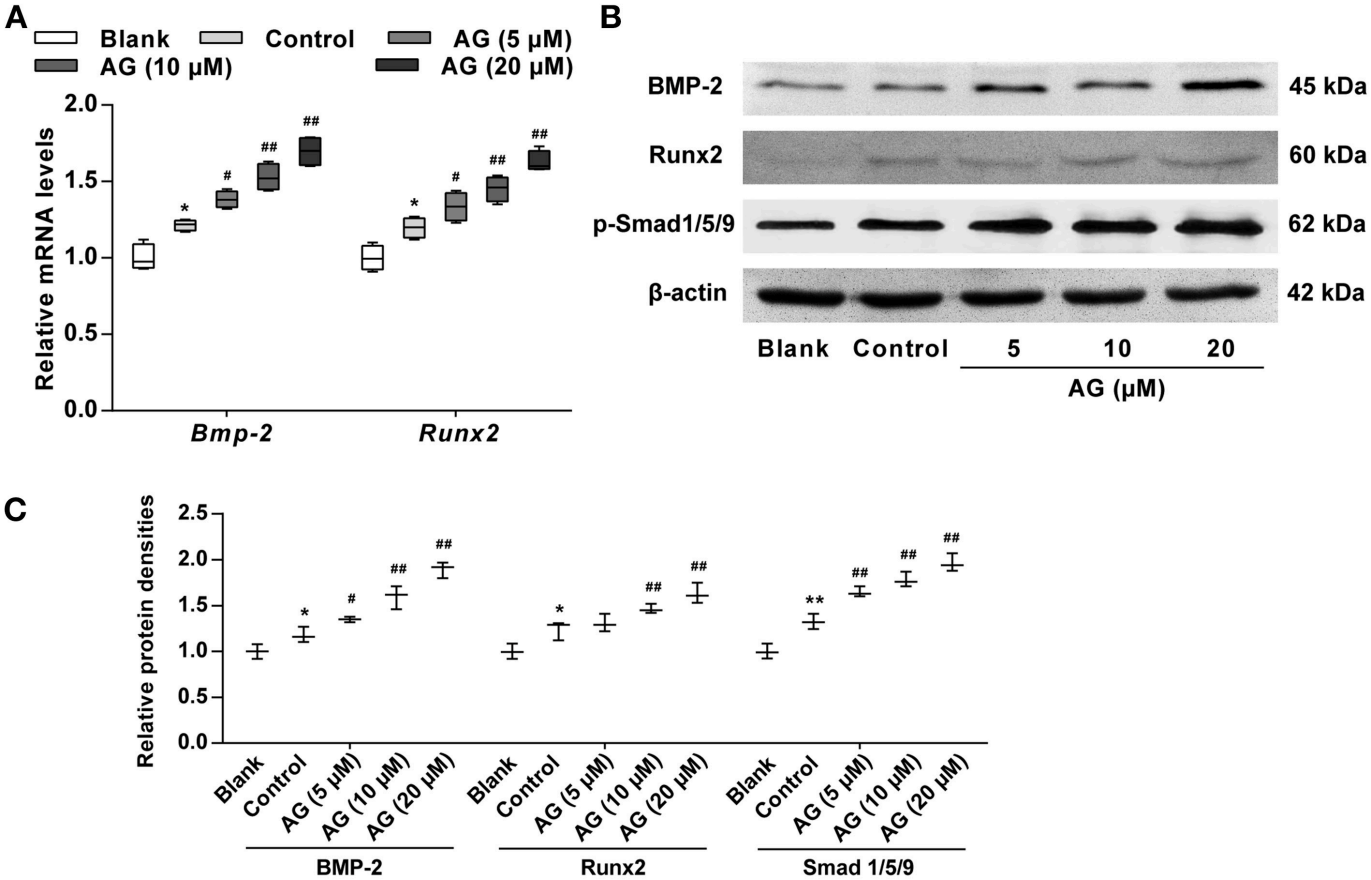
The effects of AG on the mRNA and protein levels of BMP signaling molecules in mouse MC3T3-E1 cells. MC3T3-E1 cells were treated with AG at 5, 10, and 20 μM for 24 h. **(A)** Quantitative analysis of the mRNA levels of *Bmp-2* and *Runx2* using RT-qPCR. *N* = 4. **(B)** The protein levels of BMP-2, Runx2, and p-Smad1/5/9 were determined by western blot. **(C)** Quantitative analysis of the protein levels of BMP-2, Runx2, and p-Smad1/5/9 in AG-treated MC3T3-E1 cells. *N* = 3. All of the data are shown as the mean ± S.D. of the independent experiments. ^*^P < 0.05, ^**^P < 0.01 compared with the corresponding blank group. ^#^*P* < 0.05, ^##^*P* < 0.01 compared with the corresponding control group.

Considering that the BMP signaling pathway has been confirmed to play a major role in the regulation of osteogenic differentiation, we next evaluated the effects of AG on the activation of certain signaling molecules such as BMP-2 and p-Smad1/5/9. Our data showed that the mRNA and protein levels of BMP-2 and p-Smad1/5/9 were both significantly increased in AG-treated MC3T3-E1 cells when compared with MC3T3-E1 cells cultured with OS (Figures 3A–C). Subsequently, the transcription factor Runx2, the main downstream target of BMPs, was evaluated in AG-treated MC3T3-E1 cells. The mRNA and protein levels of Runx2 were markedly elevated in AG-treated MC3T3-E1 cells compared with MC3T3-E1 cells cultured with OS (Figures 3B,C).

### AG Promotes the Activation of MAPK Signaling Molecules in MC3T3-E1 Cells

**Figure 4:**
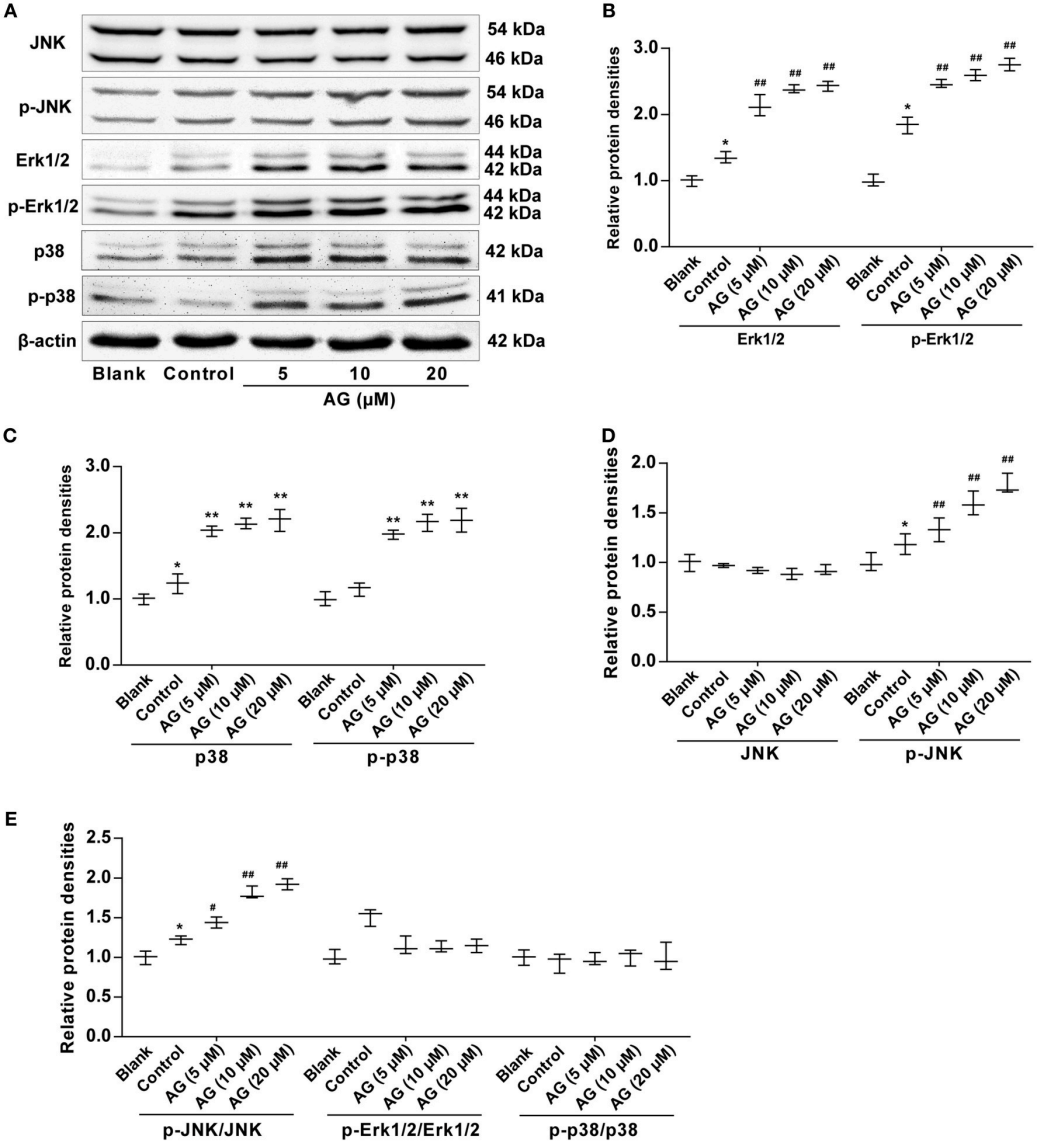
The effects of AG on the protein levels of MAPK signaling molecules in MC3T3-E1 cells. MC3T3-E1 cells were treated with AG at 5, 10, and 20 μM for 24 h. **(A)** The protein levels of p-Erk1/2, Erk1/2, p-JNK, JNK, p-p38, and p38 were measured by western blot. **(B)** Quantitative analysis of the protein levels of p-Erk1/2 and Erk1/2 in AG-treated MC3T3-E1 cells. **(C)** Quantitative analysis of the protein levels of p-p38 and p38 in AG-treated mouse MC3T3-E1 cells. **(D)** Quantitative analysis of the protein levels of p-JNK and JNK in AG-treated mouse MC3T3-E1 cells. **(E)** Quantitative analysis of the ratios of the phosphorylated proteins to the total proteins in AG-treated mouse MC3T3-E1 cells. All of the data are shown as the mean ± S.D. of the independent experiments. *N* = 3. ^*^P < 0.05, ^**^P < 0.01 compared with the corresponding blank group. ^#^*P* < 0.05, ^##^*P* < 0.01 compared with the corresponding control group.

To investigate the mechanism of AG in MC3T3-E1 cells further and to examine whether the MAPK signaling pathway was involved in the AG-induced osteoblastic differentiation of mouse MC3T3-E1 cells, we next evaluated the expression levels of MAPK signaling molecules, including extracellular signal regulated kinase (ERK), p38 MAPK kinase, and c-Jun amino-terminal kinases (JNK), by western blot. Our results demonstrated that the levels of both Erk1/2 and p-Erk1/2 were significantly increased in AG-treated MC3T3-E1 cells when compared with MC3T3-E1 cells cultured with OS (Figures 4A,B). In addition, the levels of p38 and p-p38 were also significantly increased in AG-treated mouse MC3T3-E1 cells compared with MC3T3-E1 cells cultured with OS (Figures 4A,C). Moreover, phosphorylation levels of JNK (p-JNK) and the ratio of p-JNK/JNK were significantly increased in AG-treated MC3T3-E1 cells, whereas levels of JNK remained unchanged in AG-treated MC3T3-E1 cells (Figures 4A,D,E).

### AG Promotes Bone Formation in an OVX-Induced Osteoporotic Mouse Model

**Figure 5:**
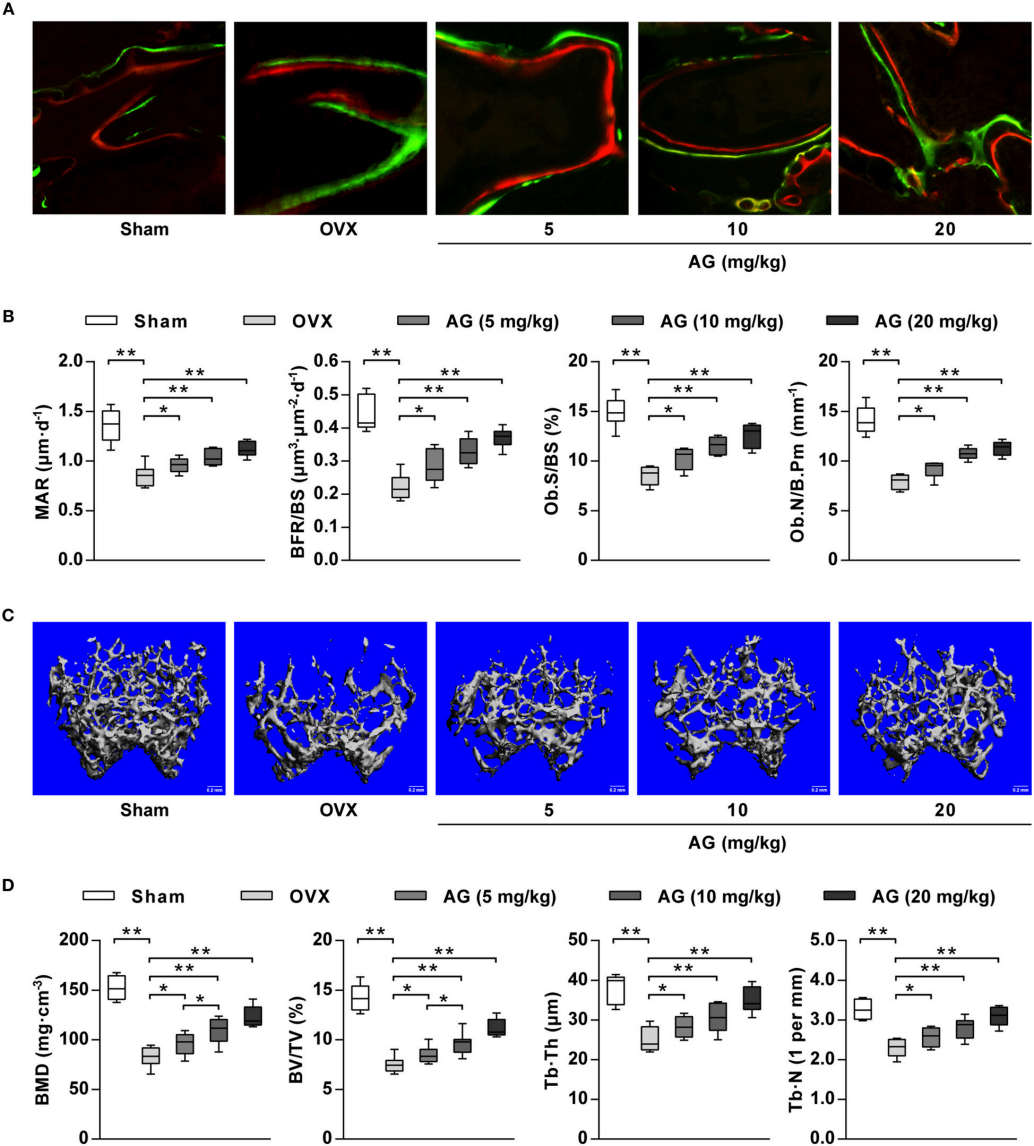
The effect of AG on bone formation *in vivo*. OVX mice were treated with different doses of AG for 4 weeks. **(A)** Representative images of bone formation at the distal femur examined using double staining with xylenol orange and calcein green in AG-treated mice and OVX mice. **(B)** The values of MAR, BFR/BS, Ob.S/BS, and Ob.N/B.Pm at the isolated distal femur from AG-treated mice and OVX mice determined using bone histomorphometry analysis. **(C)** Representative images of the distal femur metaphysis in AG-treated mice and OVX mice reconstructed using micro-CT. Scar bars, 0.2 mm. **(D)** The values of BMD, BV/TV, Tb.Th., and Tb.N. at the distal femur metaphysis in AG-treated mice and OVX mice using micro-CT. ^*^P < 0.05, ^**^P < 0.01 compared with the corresponding control group. *N* = 8 for each group.

To examine whether AG-induced osteoblastic differentiation and mineralization *in vitro* correlated with an increase in bone formation *in vivo*, an osteoporotic mouse model was constructed by ovariectomy. After 4 weeks of treatment with different doses (5, 10, and 20 mg/kg) of AG, undecalcified bone histomorphometry indicated that AG significantly increased the width between calcein green and xylenol orange labeling in the distal femur compared with the corresponding OVX group (Figure 5A). In addition, bone histomorphometric analysis indicated that both bone formation parameters and osteoblast-related parameters (BFR/BS, MAR, Ob.S/BS, and Ob.N/B.Pm) at the distal femur were significantly increased in AG-treated OVX mice compared with the OVX group, whereas no significant changes were found in osteoclast-related parameters (Oc.S/BS and Oc.N/B.Pm) at the distal femur (Figure 5B and Supplementary Figure 1). In addition, reconstructed micro-CT images demonstrated that AG attenuated the poorly organized trabecular architecture and lower bone mass at the distal femur induced by OVX (Figure 5C). Further micro-CT analysis revealed that the values of BMD, BV/TV, Tb.Th., and Tb.N. were all significantly elevated in the AG-treated groups when compared with the corresponding OVX group (Figure 5D).

## Discussion

Osteoporosis is characterized by thin and brittle bones, increasing the incidence of fractures (24). The classic triad considered in osteoporosis is severe morbidity and mortality and an enormous cost (25). Recently, the morbidity of osteoporosis has been increasing because of the change in the population structure and the increase in life expectancy. Clinical data have shown that the drugs typically used for the treatment of osteoporosis are bone resorption inhibitors (e.g., estrogen, calcitonin, and bisphosphonates) (26). The majority of these treatments serve to reduce bone fractures by inhibiting bone resorption, with the positive effects of these drugs on bone mass recovery fairly general (26). Therefore, it is urgent to find new potential candidate drugs that promote osteogenesis for the treatment of osteoporosis. AG is a flavonoid compound extracted from various traditional herbs and medicinal plants. In this study, we evaluated the promoting effect of AG on osteoblastic differentiation in mouse MC3T3-E1 cells and related mechanisms *in vitro*. In addition, we then utilized an OVX-induced osteoporosis mouse model to examine the regulatory effects of AG on *in vivo* bone formation.

Osteoblastic differentiation is a complex process that can be controlled by a great number of molecules, including ALP and Col-I (27). However, the most extensively approved biochemical markers of osteoblastic differentiation are the elevated ALP activity and the increased mineralized nodules. Indeed, the enzyme is believed to play a key role in osteoblastic differentiation and matrix mineralization (28). In this study, ALP activity was significantly increased in AG-treated cells compared with the control group. Meanwhile, matrix mineralization was also increased in AG-treated cells in a concentration- and time-dependent manner. These data demonstrated that AG might promote cellular differentiation in mouse MC3T3-E1 cells.

During the process of osteoblast differentiation, OCN and OPN are also two specific proteins present in osteoblasts. It has been reported that the levels of OCN and OPN are significantly elevated during BMP-2-induced osteoblast differentiation (6). In our study, the mRNA and protein levels of osteogenic differentiation-related molecules, including ALP, OCN, and OPN, in MC3T3-E1 cells after exposure to AG were measured by RT-qPCR and western blot, respectively. We found that AG significantly increased both the mRNA and protein levels of ALP, OCN, and OPN. These findings further confirmed that AG may serve to promote osteoblastic differentiation in MC3T3-E1 cells.

BMPs are a class of glycoproteins that play a major role in bone development and remodeling, and they are considered to be the most effective promoters of osteoblastic differentiation and bone formation (29). It has been reported that BMPs can promote chemotaxis and the aggregation of cells to osteogenic sites in different ways and promote osteoblast differentiation (30). In addition, BMPs are essential for bone development and play a major role in fracture healing (31). Among the BMP family members, BMP-2 represents a major signaling pathway for promoting bone formation. In addition, BMP-2 promotes differentiation by enhancing the activity of ALP, as well as osteocalcin and collagen synthesis (32). In this study, we found that AG increased BMP-2 protein levels, which is consistent with our RT-qPCR results.

Considering that Runx2 is the main downstream regulator of the BMP signaling pathway and it up-regulates the expression of several osteoblast-related genes (e.g., ALP, Col-I, and OPN) (33, 34), we further measured the expression of Runx2 by RT-qPCR and western blot. Our data indicated that AG significantly increased the mRNA and protein levels of Runx2 in mouse MC3T3-E1 cells. Smad1/5/9 phosphorylation is required for BMP-2 activation to stimulate the expression of Runx2 (35). In our study, we found that AG also up-regulated the protein levels of Smad1/5/9 in mouse MC3T3-E1 cells. In addition, the transcription factor p53 is involved in osteoblast differentiation by suppressing the transcription of the *RUNX2* gene (36). Thus, it is supposed that the expression of p53 could be regulated during AG-induced osteoblast differentiation in MC3T3-E1 cells. Taken together, these data revealed that AG likely promotes osteoblastic differentiation through activation of the BMP signaling pathway.

In addition, three major families of MAPKs, namely, ERK, p38, and JNK, have been reported to be associated with osteoblastic differentiation (37–39). To investigate the mechanism of AG in MC3T3-E1 cells, we further evaluated the protein levels of MAPK signaling molecules. In the present study, we found that AG significantly increased the levels of Erk1/2, p-Erk1/2, p38, p-p38, and p-JNK during the osteoblastic differentiation of mouse MC3T3-E1 cells. These data also indicated that AG might induce osteoblastic differentiation in MC3T3-E1 cells via the Erk1/2-p38-JNK-dependent signaling pathway. On the other hand, it has been reported that BMP-2 can activate two MAPKs, e.g., ERK and p38. Thus, the increased levels of p-p38 and p-ERK may have a major role in elevating BMP-2 levels and osteoblastic differentiation in mouse MC3T3-E1 cells (40). Given the above data, our results demonstrated that AG induced osteoblastic differentiation in MC3T3-E1 cells through synergistic effects involving both BMP and MAPK pathways.

For our *in vivo* study, OVX-induced osteoporosis in mice was selected as a bone repair model because it has been widely used to evaluate the bone formation of bioactive molecules and biomaterials in bones (41, 42). After treatment with AG for 4 weeks, bone histomorphometric analysis and micro-CT analysis were used to measure the effect of AG on bone formation. We found that MAR, BFR/BS, Ob.S/BS, and Ob.N/B.Pm at the distal femur were significantly increased in AG-treated mice compared with OVX mice, indicating an increase in bone formation and osteoblast number after treatment with AG in OVX mice. These findings were supported further by the observed increases in the values of BMD, BV/TV, Tb.Th, and Tb.N in AG-treated mice compared with OVX mice. In addition, it has been reported that AG showed estrogenic activity against osteoporosis and upregulated ALP activity in UMR-106 osteoblastic cells (14, 43). Taken together, these data indicated that AG could be used for osteoporosis treatment.

It has been reported that AG shows various pharmacological functions such as antioxidant, anti-inflammatory, neuroprotective, antiulcer, anti-cancer, anti-obesity, cardioprotective, and antidiabetic properties (43). These activities are accomplished by the regulation of diversified molecular targets, e.g., transcription factors (NF-κB) (44), kinases (MAPK, PI3K, Akt, and ERK) (45), enzymes (iNOS, COX-2, HK2, SOD, and GPX) (11, 46), cell adhesion proteins, inflammatory cytokines, and apoptotic and antiapoptotic proteins (43). Therefore, it is possible that AG could be a potent drug candidate through structural optimization or the synthesis of more effective analogs.

However, there are some limitations to this study. First, to be consistent with the *in vitro* study, the expression levels of the osteoblast-related markers (ALP, Col-I, and OPN) and signaling molecules (ERK, p38, and JNK) should be examined by western blotting or immunohistochemical methods in *in vivo* studies. Second, we have no further research about the effect of AG on collagen metabolism. Third, the *in vivo* concentration of AG in mice, especially the concentration in the bones, is difficult to determine. However, from published data we estimate the concentration in the blood to be ~9.4 μM, which is within the range that we used in our *in vitro* experiments.

In summary, this study demonstrated that AG promotes osteoblastic differentiation and bone formation through synergistic effects involving both BMP and MAPK pathways. These findings indicate that AG may prove to be a useful bone anabolic agent for the prevention and treatment of osteoporosis.

## Ethics Statement

This study was carried out in accordance with the recommendations of the NIH guidelines for the care and use of laboratory animals. The protocol was approved by the Committees of Animal Ethics and Experimental Safety of Binzhou Medical University.

## Author Contributions

DL supervised the whole project. LL, DW, and DL performed the major research and wrote the manuscript with equal contributions. YQ, MX, LZ, WX, XL, and LY provided technical support. SY and QZ provided their professional expertise.

### Conflict of Interest Statement

The authors declare that the research was conducted in the absence of any commercial or financial relationships that could be construed as a potential conflict of interest.

## Supplementary Material

Supplementary Figure 1 The effect of AG on osteoclast-related parameters at the distal femur in mice. The values of **(A)** Oc.S/BS and **(B)** Oc.N/B.Pm at the isolated distal femur from AG-treated mice and OVX mice were determined using bone histomorphometry analysis. *N* = 8 for each group.

Data Sheet 1 Raw data for all the real-time PCR analysis.

Data Sheet 2 The uncropped western blot scans.

## Abbreviations

AG: astragalin
ALP: alkaline phosphatase
BMP: bone morphogenetic protein
Runx2: Runt-related transcription factor 2
Erk1/2: extracellular signal regulated protein kinases 1 and 2
JNK: c-Jun N-terminal kinase
MAPK: mitogen-activated protein kinase
OCN: osteocalcin
OPN: osteopontin
TGF-β: transforming growth factor-β
FBS: fetal bovine serum
MTT, 3-(4,5-Dimethylthiazol-2-yl)-2: 5-diphenyltetrazolium bromide
DMSO: dimethyl sulphoxide
EDTA: ethylenediaminetetraacetic acid
OVX: ovariectomized.
