# Supplementary figures and images for: Astragalin Promotes Osteoblastic Differentiation in MC3T3-E1 Cells and Bone Formation in vivo
Source: Front Endocrinol (Lausanne). 2019 Apr 16;10:228. doi: 10.3389/fendo.2019.00228 (PMC6476984; doi:10.3389/fendo.2019.00228)

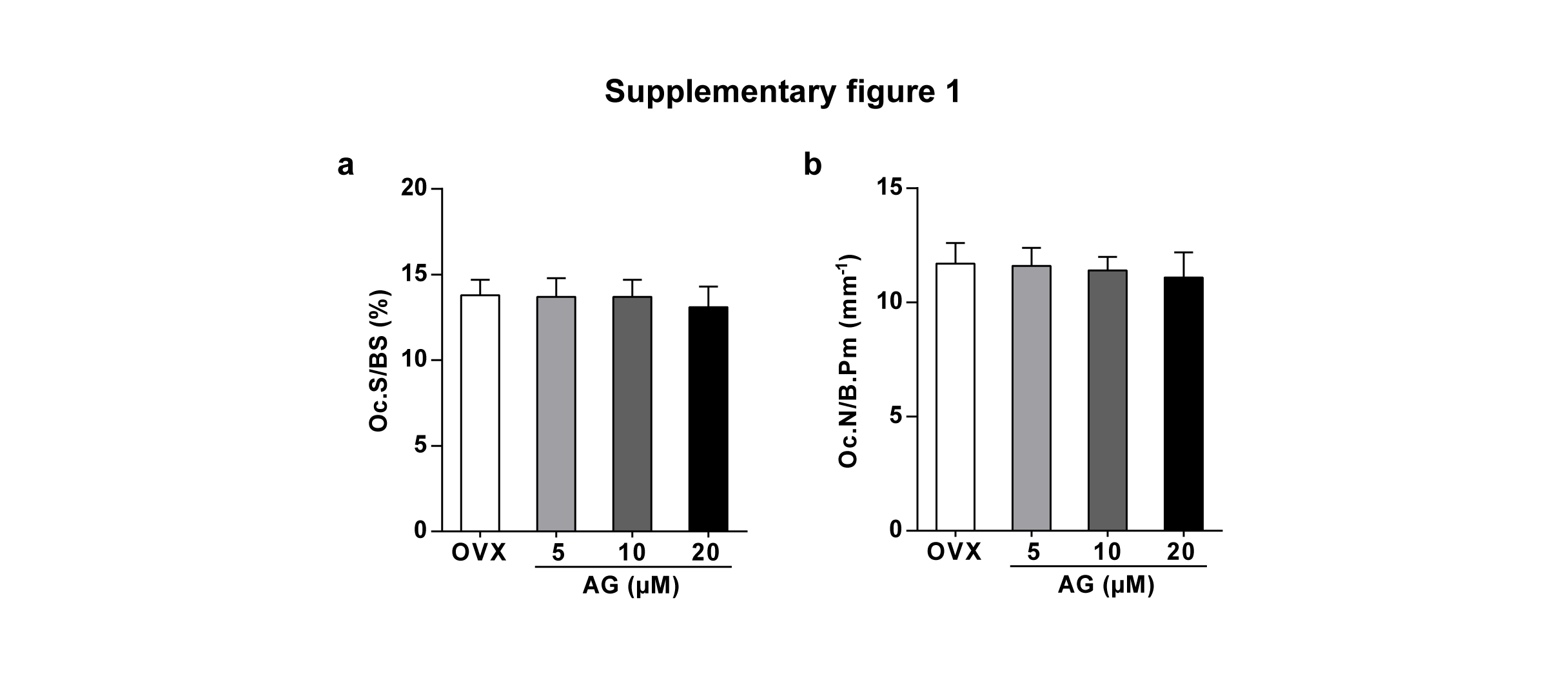

Supplement: Supplementary Figure 1 — The effect of AG on osteoclast-related parameters at the distal femur in mice. The values of (A) Oc.S/BS and (B) Oc.N/B.Pm at the isolated distal femur from AG-treated mice and OVX mice were determined using bone histomorphometry analysis. N = 8 for each group. [file Image_1.tif]
