# Supplementary material for: Astragalin Promotes Osteoblastic Differentiation in MC3T3-E1 Cells and Bone Formation in vivo
Source: Front Endocrinol (Lausanne). 2019 Apr 16;10:228. doi: 10.3389/fendo.2019.00228 (PMC6476984; doi:10.3389/fendo.2019.00228)
Supplement: Data Sheet 1 — Raw data for all the real-time PCR analysis. [file Data_Sheet_1.pdf]

Supplementary data 1 for Figure 2a

Raw data for Figure 2a

Groups (n=4)		$2^{-\triangle\triangle CT}$				Average	SD	*P*
Alp mRNA	Blank	1.06	0.99	0.87	1.08	1.000	0.095	
	Control	1.34	1.19	1.31	1.23	1.268	0.069	0.004 (vs Blank)
	AG (5 μM)	1.36	1.52	1.33	1.42	1.408	0.084	0.042 (vs Control)
	AG (10 μM)	1.55	1.5	1.57	1.59	1.553	0.039	<0.01 (vs Control)
	AG (20 μM)	1.61	1.78	1.58	1.70	1.668	0.091	<0.01 (vs Control)
Ocn mRNA	Blank	1.05	1	0.91	1.06	1.005	0.069	
	Control	1.19	1.18	1.21	1.14	1.180	0.029	0.003 (vs Blank)
	AG (5 μM)	1.29	1.37	1.26	1.24	1.290	0.057	0.014 (vs Control)
	AG (10 μM)	1.34	1.41	1.42	1.35	1.380	0.041	<0.01 (vs Control)
	AG (20 μM)	1.53	1.51	1.44	1.43	1.478	0.050	<0.01 (vs Control)
Opn mRNA	Blank	0.99	1.05	0.92	1.04	1.000	0.059	
	Control	1.19	1.14	1.23	1.22	1.195	0.040	0.002 (vs Blank)
	AG (5 μM)	1.36	1.2	1.35	1.34	1.313	0.075	0.033 (vs Control)
	AG (10 μM)	1.43	1.48	1.49	1.51	1.478	0.034	<0.01 (vs Control)
	AG (20 μM)	1.65	1.79	1.63	1.77	1.710	0.082	<0.01 (vs Control)

Raw data for Figure 3c

Groups		No OC	WT	OC-miR-214-3p	OC-miR-214-3p+ Veh	OC-miR-214-3p+ 3’UTR-NC	OC-miR-214-3p+ ATF4 mRNA 3’UTR
ALP mRNA	△CT	(-6.44)±0.21	(-7.11)±0.21	(-7.89)±0.29	(-7.83)±0.22	(-7.85)±0.33	(-7.13)±0.24
	$2^{-\triangle\triangle CT}$	1.00±0.14	0.63±0.09	0.37±0.07	0.38±0.06	0.38±0.08	0.62±0.10
OPN mRNA	△CT	(-8.83)±0.21	(-9.42)±0.19	(-10.44)±0.35	(-10.41)±0.34	(-10.34)±0.36	(-9.44)±0.19
	$2^{-\triangle\triangle CT}$	1.00±0.15	0.67±0.09	0.33±0.08	0.34±0.08	0.36±0.09	0.66±0.08
BSP mRNA	△CT	(-9.34)±0.22	(-9.90)±0.26	(-10.47)±0.24	(-10.46)±0.29	(-10.43)±0.22	(-9.94)±0.28
	$2^{-\triangle\triangle CT}$	1.00±0.15	0.68±0.12	0.46±0.07	0.46±0.09	0.47±0.07	0.67±0.13
BGLAP mRNA	△CT	(-6.95)±0.14	(-7.58)±0.26	(-8.45)±0.36	(-8.41)±0.35	(-8.42)±0.47	(-7.55)±0.13
	$2^{-\triangle\triangle CT}$	1.00±0.09	0.65±0.11	0.36±0.09	0.37±0.10	0.38±0.12	0.66±0.06

Raw data for Figure 3i

Groups		No OC	WT OC	OC-miR-214-3p
miR-214-3p	△CT	(-12.10)±0.57	(-3.87)±0.24	(-2.13)±0.16
	$2^{-\triangle\triangle CT}$	0.00±0.00	1.00±0.17	3.33±0.38

Raw data for Figure 3j

Groups		No OC	WT osteoclasts	miR-214-3p-depleted OCs
Pri-miR-214-3p in osteoblasts	△CT	(-5.28)±0.17	(-5.21)±0.22	(-5.25)±0.26
	$2^{-\triangle\triangle CT}$	1.00±0.12	1.05±0.15	1.03±0.19
Pre-miR-214-3p in osteoblasts	△CT	(-4.68)±0.26	(-4.64)±0.25	(-4.65)±0.19
	$2^{-\triangle\triangle CT}$	1.00±0.17	1.03±0.17	1.01±0.13
miR-214-3p in osteoblasts	△CT	(-3.16)±0.17	(-1.89)±0.08	(-2.70)±0.14
	$2^{-\triangle\triangle CT}$	1.00±0.12	2.40±0.13	1.37±0.13

Raw data for Figure 4c

Groups		WT-Exo	miR-214-3p-Exo
Pri-miR-214-3p in osteoblasts	△CT	(-5.41)±0.27	(-5.31)±0.23
	$2^{-\triangle\triangle CT}$	1.00±0.19	1.07±0.16
Pre-miR-214-3p in osteoblasts	△CT	(-4.79)±0.23	(-4.73)±0.24
	$2^{-\triangle\triangle CT}$	1.00±0.16	1.04±0.17
miR-214-3p in osteoblasts	△CT	(-3.45)±0.30	(-2.17)±0.19
	$2^{-\triangle\triangle CT}$	1.00±0.21	2.39±0.33

Raw data for Figure 4d

**Groups**		WT-Exo	miR-214-3p-Exo
ALP mRNA	△CT	(-6.72)±0.22	(-7.87)±0.33
	$2^{-\triangle\triangle CT}$	1.00±0.15	0.46±0.10
OPN mRNA	△CT	(-9.17)±0.24	(-10.17)±0.46
	$2^{-\triangle\triangle CT}$	1.00±0.17	0.52±0.15
BSP mRNA	△CT	(-8.87)±0.27	(-9.57)±0.37
	$2^{-\triangle\triangle CT}$	1.00±0.19	0.63±0.15
BGLAP mRNA	△CT	(-7.36)±0.23	(-8.23)±0.29
	$2^{-\triangle\triangle CT}$	1.00±0.16	0.55±0.10

Raw data for Figure 5b

**Groups**		OVX-BL	OVX	OVX+Veh	OVX+NC	OVX+AMO	Sham
miR-214-3p in osteoclasts	△CT	(-1.77)±0.31	(-0.14)±0.16	(-0.11)±0.17	(-0.10)±018	(-2.51)±0.47	(-3.10)±0.67
	$2^{-△△CT}$	1.00±0.20	3.06±0.32	3.12±0.34	3.14±0.36	0.62±0.20	0.42±0.17
miR-214-3p in osteoblasts	△CT	(-5.41)±0.32	(-4.49)±0.14	(-4.45)±0.16	(-4.46)±0.14	(-6.19)±0.36	(-7.12)±0.66
	$2^{-△△CT}$	1.00±0.21	1.86±0.18	1.92±0.21	1.89±0.18	0.59±0.14	0.32±0.12
