# Supplementary material for: Astragalin Promotes Osteoblastic Differentiation in MC3T3-E1 Cells and Bone Formation in vivo
Source: Front Endocrinol (Lausanne). 2019 Apr 16;10:228. doi: 10.3389/fendo.2019.00228 (PMC6476984; doi:10.3389/fendo.2019.00228)

Supplementary data 2 for western blots

Raw data for Figure 2b

ALP

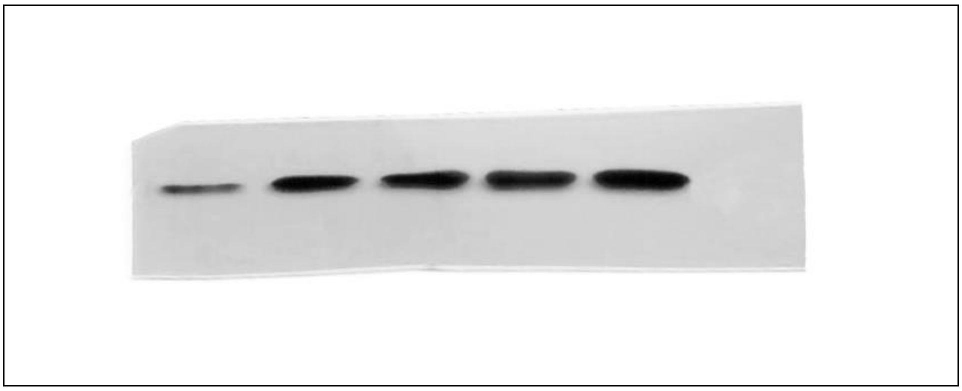

OCN

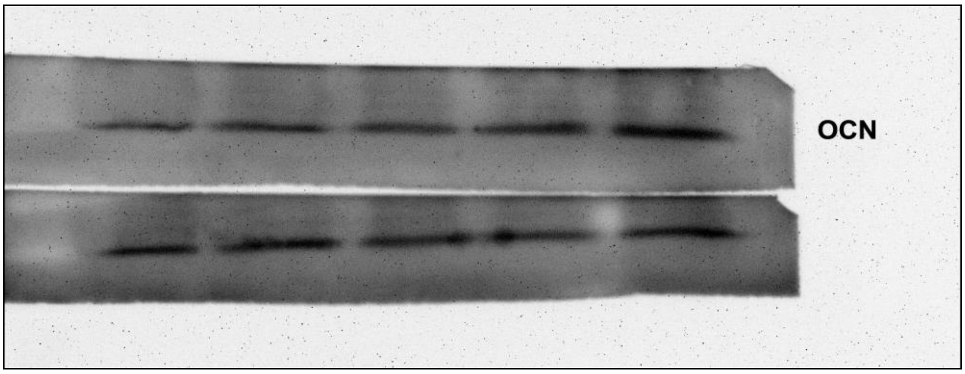

OPN

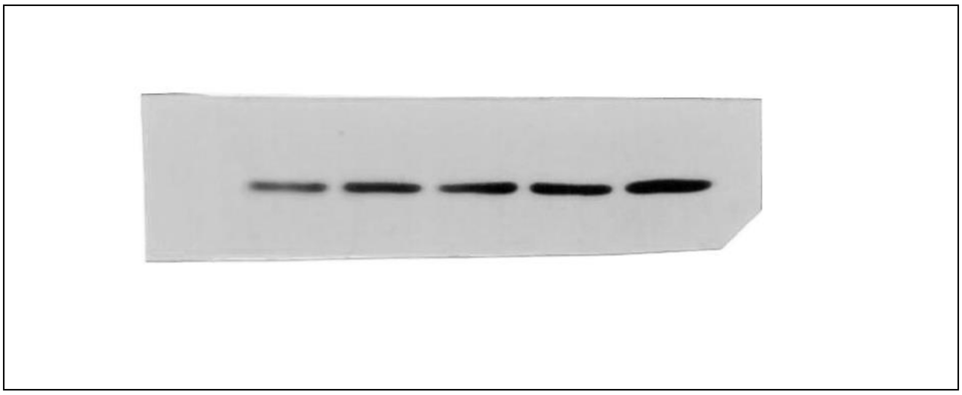

β-actin

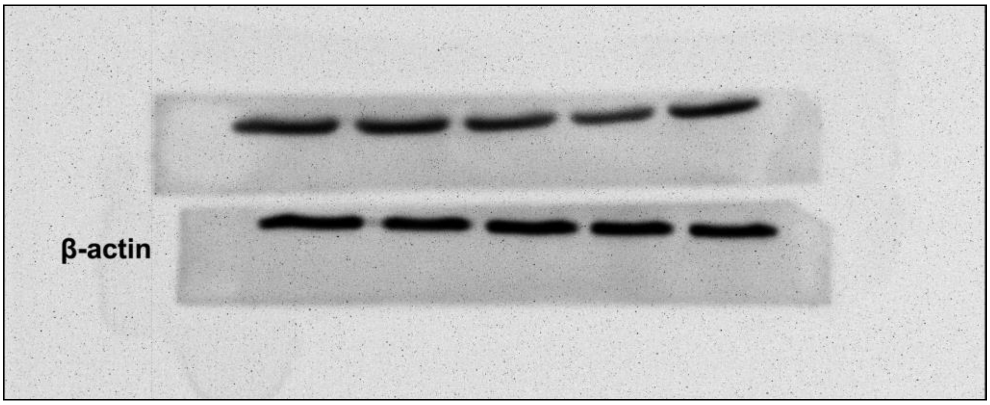

Raw data for Figure 3b

BMP-2

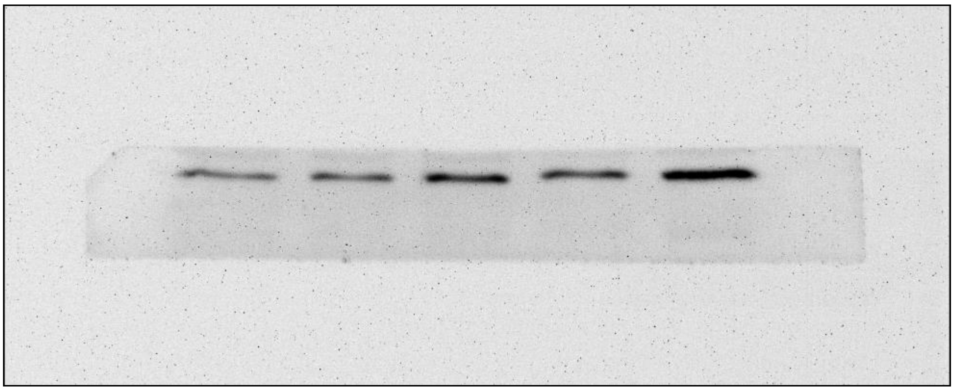

Runx-2

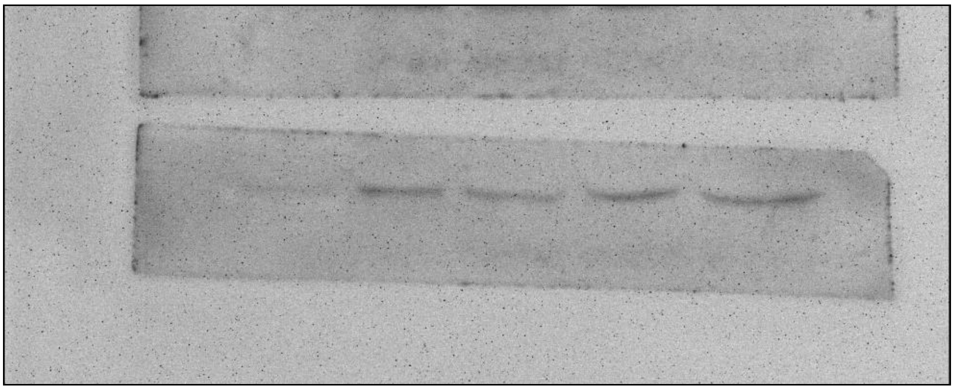

β-Smad1/5/9

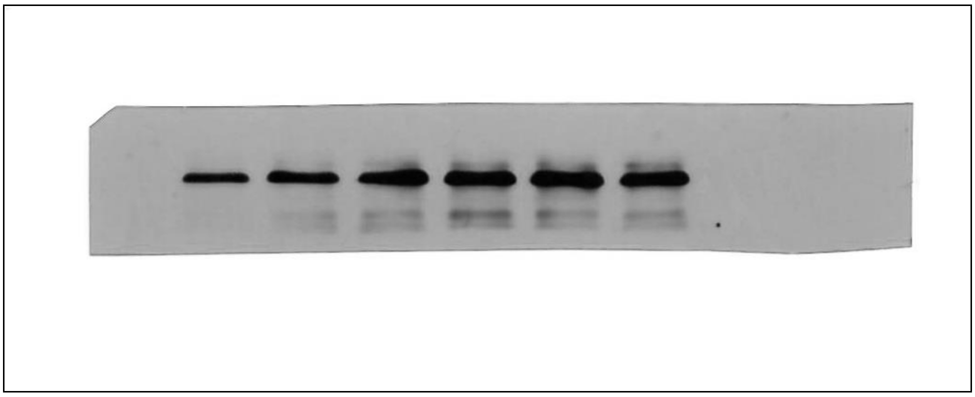

β-actin

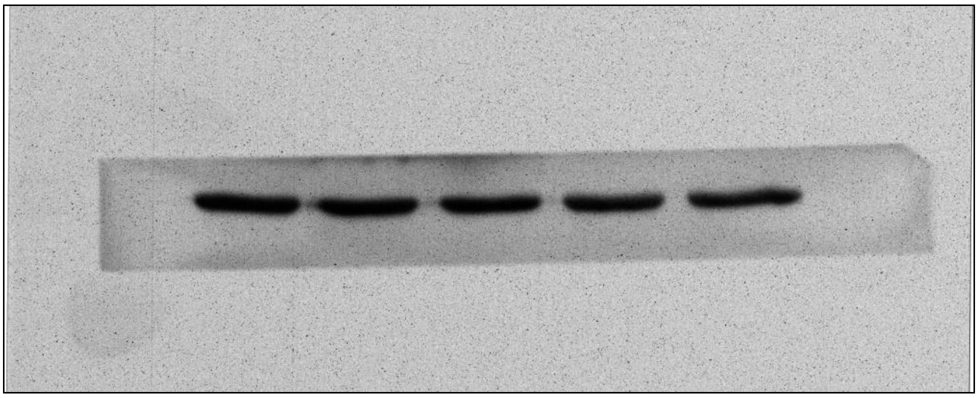

Raw data for Figure 4a

JNK

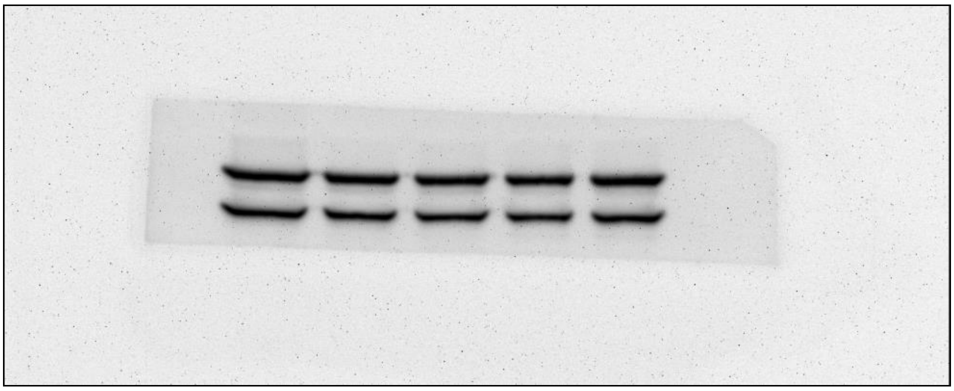

p-JNK

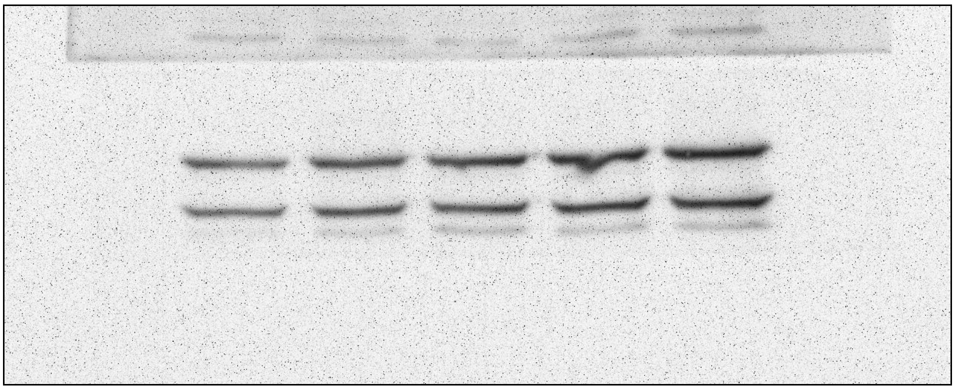

Erk1/2

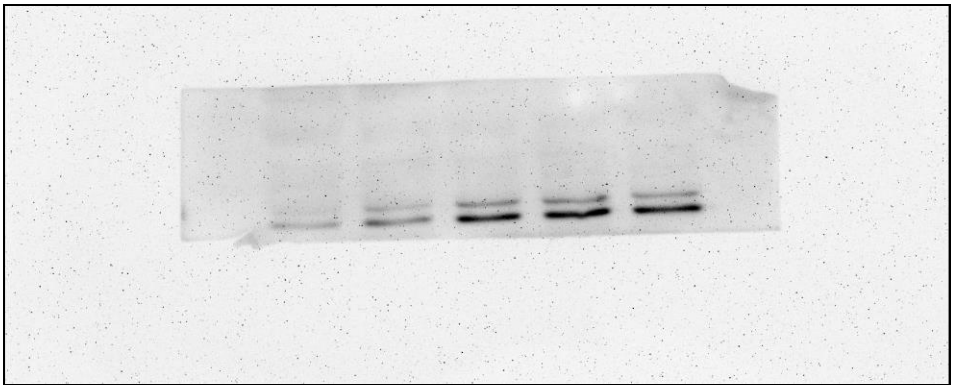

p-Erk1/2

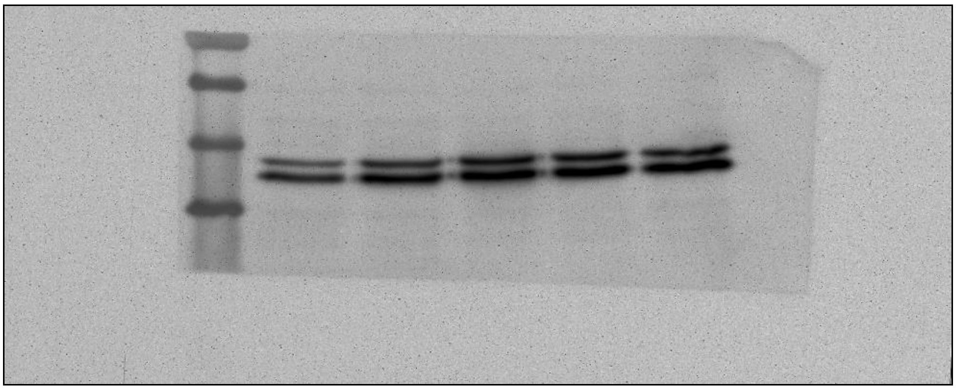

p38

p-p38

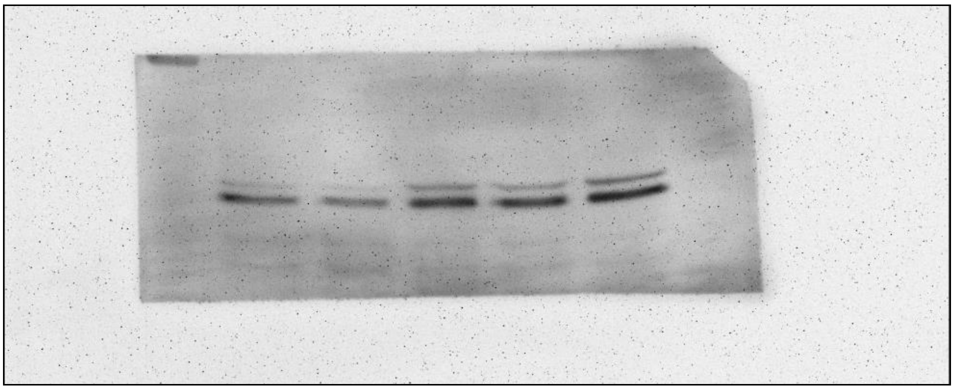

β-actin

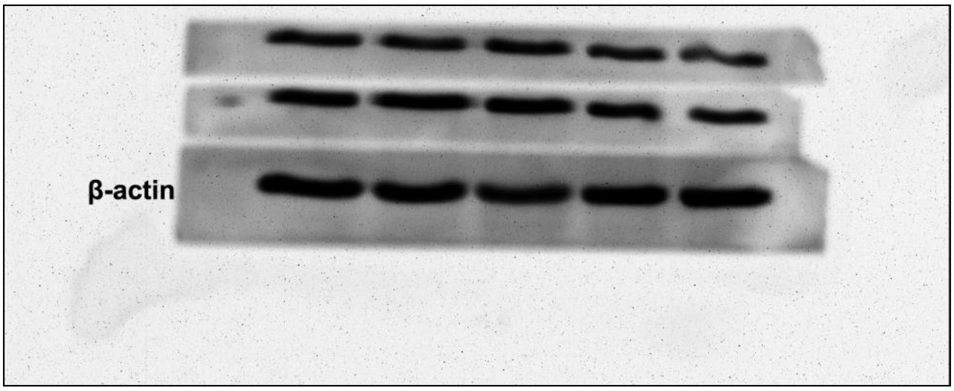

Supplement: Data Sheet 2 — The uncropped western blot scans. [file Data_Sheet_2.pdf]
